# SUPERVISED EXERCISE AFTER MINOR STROKE: AN EVALUATION FROM THE PERSPECTIVE OF PATIENTS AND HEALTHCARE PROFESSIONALS

**DOI:** 10.2340/jrm.v57.42881

**Published:** 2025-03-24

**Authors:** Rikke Steen KRAWCYK, Katrine Vollbrecht AMDI, Christina KRUUSE, Thordis THOMSEN

**Affiliations:** 1 Department of Physiotherapy and Occupational Therapy, Copenhagen University Hospital – Herlev and Gentofte, Copenhagen, Denmark; 2 Department of Neurology, Neurovascular Research Unit, Copenhagen University Hospital – Herlev and Gentofte, Copenhagen, Denmark; 3 Department of Brain and Spinal Cord Injury, Neuroscience Center, Copenhagen University Hospital Rigshospitalet, Copenhagen, Denmark; 4 Department of Clinical Medicine, University of Copenhagen, Copenhagen, Denmark; 5 Department of Anaesthesiology, Copenhagen University Hospital – Herlev and Gentofte, Copenhagen, Denmark

**Keywords:** behaviour change, cross-sectoral intervention, minor stroke, physical exercise, physiotherapy, qualitative research, stroke prevention, transient ischaemic attack

## Abstract

**Objective:**

Maintaining long-term physical activity after a stroke is challenging. “The Stroke School”, a standardized physical exercise programme, was developed and patients’ and healthcare professionals’ experiences of participating were explored.

**Design:**

Qualitative study.

**Methods:**

Eight patients with minor stroke or transient ischaemic attack (TIA) completed a feasibility study on The Stroke School intervention in conjunction with their supervising municipal physiotherapists (*n* = 5). All informants were invited for semi-structured focus-group interviews, during which they were asked to reflect on their experience attending The Stroke School. Audio recordings from 3 focus-group interviews lasting 90 min each were transcribed to text verbatim and analysed with qualitative content analysis.

**Results:**

Thirteen informants attended 3 focus-group interviews. Five categories were identified (*i–iii* representing the patients’ perspective and *iv–v* the physiotherapists’ perspectives): (*i*) window of opportunity, (*ii*) benefits of participating in the study, (*iii*) strengths and pitfalls of transitioning from the hospital to the municipalities, (*iv*) effective communication across sectors, and (*v*) empowering patients to continue exercising independently.

**Conclusion:**

The Stroke School intervention was feasible, provided patient safety throughout the study, and resulted in effective communication and collaboration across sectors. However, identifying factors that facilitate life-long exercise behaviour changes is still warranted.

## LAY ABSTRACT

Patients with non-disabling stroke do not systematically receive a physical exercise programme following hospital discharge as their symptoms are often resolved at this point. However, initiating and maintaining physical activity independently to reduce stroke risk factors can be challenging post-stroke due to fatigue, reduced mental energy, and cognitive dysfunction. We designed a supervised exercise study (The Stroke School), initiated before hospital discharge. Furthermore, we explored patients’ and healthcare professionals’ experiences of participating in the supervised physical exercise programme. The results showed that the exercise intervention helped to ensure patient safety through frequent exercise sessions, follow-up examinations, and repeated counselling sessions. Patients were highly motivated to exercise immediately after their stroke diagnosis. The municipal physiotherapists experienced effective communication and collaboration across sectors but empowering the patients to exercise independently after ending 12 weeks of supervised exercise was challenging.

Evidence shows that it cannot be determined whether exercise reduces mortality or the chances of death or dependency in patients with stroke (1). However, physical activity, specifically cardiorespiratory exercise, is generally recommended to reduce disability and increase mobility and physical function (balance) in patients with stroke (2). A sub-group of stroke patients, those with minor stroke and transient ischaemic attack (TIA), do not systematically receive a standardized physical exercise programme following hospital discharge as their symptoms are often resolved at this time. The usual treatment for this group of patients is medical secondary prevention and suggestions for self-administered lifestyle changes. Studies, however, show that initiating and maintaining a sufficient level of physical activity is challenging for patients with minor strokes due to post-stroke fatigue, reduced mental energy, and cognitive or physical dysfunction (3, 4). Previous studies have shown that an 8-week, twice-weekly supervised exercise programme for patients with minor stroke improved self-perceived health (5) and vascular risk factors (systolic blood pressure and aerobic fitness [VO_2peark_]) (6). Our previous research identified 4 essential motivators for physical activity after hospital discharge in patients with minor stroke or TIA; (*i*) being able to exercise close to home; (*ii*) supervised exercise; (*iii*) exercising with peers; and (*iv*) making the exercise a weekly habit (7). Based on this, we designed and evaluated a standardized physical exercise programme, “The Stroke School” in an initial feasibility study. This study aimed to explore patients’ and healthcare professionals’ experiences of participating in the standardized physical exercise programme. The results from this qualitative study together with the results of the initial feasibility study will inform the design of a subsequent larger randomized controlled trial (8).

## METHODS

### Study design

A pragmatic qualitative study with focus-group interviews was undertaken. Focus groups were chosen as they encouraged informants to share experiences with peers (9). The study was reported according to the Consolidated Criteria for Reporting Qualitative Studies (COREQ checklist), using the items relevant to a pragmatic qualitative study (10).

### Settings

All focus-group interviews were conducted face-to-face in an undisturbed room at a large tertiary hospital in Copenhagen, Denmark, and lasted approximately 90 min. The only individuals in the room were the researchers (RSK and KVA), and the informants.

### The Stroke School intervention

Eligibility criteria for The Stroke School: (*i*) a diagnosis of minor ischaemic stroke or TIA according to the International Classification of Diseases codes I63: cerebral infarction, and G45: transient ischaemic attack. Minor stroke was defined as a stroke with low symptom severity (National Institute of Health Stroke Scale [NIHSS ≤ 4 points]); TIA as symptom remission within 24 h; (*ii*) patients not requiring subsequent rehabilitation but in need of an exercise plan to prevent recurrence (evaluated by therapists during hospital stay); (*iii*) patients who had participated in ≤ 5h of vigorous-intensity activity every week within the last 3 months. The Stroke School was an outpatient group intervention encompassing 6 weeks of cardiovascular exercise at the hospital (initiated closely after hospital discharge) and continued for an additional 6 weeks within the local municipality. The intervention consisted of cardiovascular exercise (cycling) twice a week lasting 1 h. Health professionals actively participated in the hospital training, cycling alongside the patients. In the municipal phase, the patients joined pre-existing cardiovascular exercise groups doing cardiovascular exercise and strength training (1 h twice weekly). The exercises for cardiac patients were identical to those recommended for patients with minor stroke. The municipal physiotherapists supervised the sessions, however, they did not actively engage in physical exercise. Throughout the 12 weeks of intervention, patients attended one-on-one patient counselling (2 sessions of an hour’s duration at the hospital) with the primary researcher. The focus was on lifestyle factors. Additionally, the patients participated in 3 individual follow-up sessions, lasting an hour, with the local municipal physiotherapist. The first session was conducted face-to-face immediately after terminating the last exercise session; the remaining follow-up sessions were conducted by telephone between 3 and 6 months after stroke onset. The focus of the municipal follow-up sessions was on how to consolidate long-term behavioural change to being physically active.

### Recruitment

We recruited 2 groups of informants using convenience sampling: 1 group consisted of patients with minor stroke or TIA who had completed The Stroke School intervention and the other of municipal physiotherapists who supervised the exercise. All informants (patients and municipal physiotherapists) were invited by email. We planned for 3 focus-group interviews (2 with patients and 1 with municipal physiotherapists) with 4–8 informants in each focus-group interview, leaving room for dropouts without compromising group dynamics and the possibility for everybody to express their opinion (11).

### Research team: personal characteristics

RSK is a female researcher (PhD) with 23 years of clinical experience as a physiotherapist, 18 years of which are in neurorehabilitation. KVA is a female nurse with a Master’s degree and 8 years of clinical experience working with stroke patients. Both RSK and KVA have previous experience in conducting qualitative research. TT and CK are female professors with over 25 years of clinical experience. TT is a clinical professor and nurse in anaesthesiology and has extensive experience in qualitative research, and CK is a medical doctor and professor in stroke treatment with moderate experience in qualitative research. RSK and KVA had prior knowledge of the patients as they had supervised them during exercise and assessed outcome measures. Thus, it was decided *a priori* that the individual who had examined and exercised with patients in the respective focus groups would act as an observer.

### Data collection

KVA moderated the first focus-group interview with patients, while RSK moderated the second. RSK also moderated the focus-group interview with municipal physiotherapists. All informants were aware of the purpose of the study and the researcher’s interest in exercise behaviour change.

### Interview guide

Two semi-structured interview guides (Table I), 1 for the municipal physiotherapists and 1 for patients, guided the focus-group interviews. The interview guides were based on the feasibility study purpose, scientific literature on the subject (1, 7), and discussion in the research group. The interview guide was not pilot-tested before the study commencement but adjusted during data collection. During the process, 1 question was add-ed on their experience of exercising independently. All focus -group interviews started with an introduction, including a presentation of the study’s purpose and informants. Also, the two researchers’ roles were clarified, and the confidentiality concern was highlighted.

**Table I:** Interview guide for the municipal physiotherapists and for the patients

Main questions		Probes (optional)
**Municipal physiotherapists**		
1	Please describe what it was like to be a part of this cross-sectoral exercise study	Please describe your general experience. Was it exciting to be a part of the study or was there too much work? Can we reduce your workload? Are there any issues that need to be addressed before we can proceed with an RCT?
2	Please describe how you experienced the logistical transition for the individual patient from exercising in the hospital to exercising in the municipality	Has there been a delay in patients switching between sectors? Is the standard electronic reference received on time? Is it sufficient? Are patients automatically assigned to exercise with the cardiac rehabilitation group? Are patients offered an initial meeting before exercising in the municipality?
3	Please describe the manner in which you experienced the communication between the study researchers and yourself, including the types of verbal, telephone, and email communication you engaged in	Please describe your experience of the information provided prior to patient enrolment. Please indicate whether you felt that you lacked sufficient information about the study. Please also indicate whether you could contact the researcher by telephone or email if you have any further questions.
4	Please describe your experience of including patients with stroke in your cardiac rehabilitation programme	To what extent do you feel confident in exercising with patients with stroke in terms of time and resources? Have you been successful in integrating stroke patients into a cardiac rehabilitation group?
5	Please describe your experience with the individual follow-up sessions	What difficulties are encountered in the individual follow-up sessions?
6	Please describe your experience with the REDcap documentation programme	Please describe your experience of REDcap in terms of time, content, and extent. Have you encountered any difficulties when working with REDcap? Please evaluate the ease of use. How was REDcap introduced to municipal physiotherapists before study enrolment?
**Patients**		
1	Please describe what it was like to be a part of this exercise study	Please describe your thoughts when invited to participate in the study, including any considerations you took into account when deciding to participate. Please describe whether you found the study attractive and, if so, what aspects of it were appealing to you. Please describe your thoughts about the cardiovascular exercise intervention, including whether you found it meaningful and what you expected to gain from participating in the study.
2	What are your views on the merits of undertaking cardiovascular exercise after a stroke?	Have you experienced concerns regarding the feasibility of exercise following a stroke? How has the experience of supervised cardiovascular exercise been for you? Has your participation in this study altered your perspective on the value of cardiovascular exercise? Do you perceive any changes in your mental or physical state following the initiation of exercise? Have you received support from your family members regarding your exercise regimen?
3	Please describe the changes that occurred when you transi-tioned from exercising at the hospital to the municipality	Please describe the temporality of the transition from exercising at the hospital to the municipality. Please describe the municipality’s welcoming environment.
4	Please describe your experience of the exercise sessions at the hospital	Please provide your opinion on the timing of the first exercise session. Do you have any comments on the scheduled time for the sessions? Please describe your experience of the atmosphere during exercise. Please also provide your opinion on the intensity of the exercise.
5	Please describe the supervised exercise sessions in the municipality, including the experience of exercising together with patients with cardiac problems	Please describe the timing of the initial exercise session in the municipality. Do you have any comments regarding the scheduled time for the sessions? Please describe your experience of exercising with patients in the cardiac rehabilitation group. Please indicate whether you felt that you were a part of the cardiac rehabilitation group.
6	Please elaborate on your experience of exercising independently	Please describe any challenges you may have encountered when exercising independently. Additionally, please indicate whether you felt any sense of insecurity or unsafe conditions when exercising on your own. Please describe your motivation for exercising independently.
7	Please describe your experience of the maximal cardiopulmonary exercise test	Please describe your experience of wearing a face mask during the test. Please indicate whether you had any concerns before the test. Please describe whether you were able to push yourself to your maximum capacity on the first and second occasions.
8	Please share your views on the chosen outcomes in terms of patient relevance	Are the selected outcomes meaningful to you as a patient? Would the study benefit from the inclusion of supplementary patient-reported outcomes? What do you as a patient hope to gain from involvement in the study?
9	Please describe your experience of the individual follow-up sessions conducted in the municipality	Please describe how we can support you in maintaining your exercise routine after study termination
10	Please describe your experience of the individual stroke prevention sessions	Did you find the lifestyle information useful? Anything in the stroke prevention section you would like to see covered in more detail?

The interviewer sought to cover the main questions, whereas probes were optional, and meant as a help for the interviewer.

### Data analysis

The interviews were audio recorded, and the audio files were immediately transferred to a computer with a safe drive, according to current legislation (the Danish Protection Agency). The audio files were transcribed verbatim and analysed using content analysis as described by Elo and Kyngäs (12). The transcribed interviews were not sent to the informants for corrections and approval. The qualitative content analysis had an inductive approach, first deriving codes from meaning units, grouping, and creating sub-categories and categories from the data (12) (Table II). Before the coding process, RSK and KVA familiarized themselves with the data and analysed 5 pages of the first interview together to align the analysis process. Following this, RSK and KVA analysed data in parallel. Finally, RSK and KVA refined categories and sub-categories and translated them into English. Investigator triangulation was achieved through active involvement of multiple researchers with different professional backgrounds contributing to the analysis. These included a medical doctor, a physiotherapist, and nurses. RSK and KVA did the initial coding of data. CK and TT were subsequently actively involved in the further condensation of data and formulation of categories (13). The overall findings and data saturation were discussed and there was consensus in the entire research group that data saturation was achieved (RSK, KVA, TT, and CK). QSR Nvivo software (version 14.23.1.38; https://lumivero.com/products/nvivo/) was used to organize the data analysis, to verify and validate transactions by tracking user activity and thereby reduce the risk of material errors and unauthorized use. Microsoft Excel 2016 (Microsoft Corporation, Redmond WA, USA) was used to describe baseline characteristics.

**Table II:** Example of coding tree

Meaning unit/quotes	Condensation	Sub-theme	Theme
“… what is nice (about being a part of this study) is all the follow-ups … it is also something my family appreciates … it provides security for the ones at home.” “Yes I was worried in the beginning about increasing my heart rate … I was afraid that being physically active would result in a recurrent stroke…. That is all history now.”	Supervised exercise and regular follow-ups provide safety for the patients and their families	Safety throughout the study period	Benefits of participating in the study
” We share the experience of having a stroke…. [I liked] having the opportunity to talk [with peers] for 5–10 minutes before or/and after the exercise sessions. To talk about our experiences with everyday life with stroke.” “I was allowed to bring my husband [to the exercise sessions] … as I was the only patient [included for exercise in the study] at the time … and it was super cool.”	The patients profited socially from exercising with others. It was obligating being part of a group with a light and moody atmosphere	Sharing and bonding with peers	

### Ethical considerations

The study was approved by the regional Committee on Health Research Ethics in the Capital Region of Denmark (ID: H-20059985) and the Danish Data Protection Agency (ID: P-2020-1088). All informants received written and oral information concerning the study and provided written informed consent before study enrolment according to the Helsinki Declaration (14).

## RESULTS

**Fig. 1:**
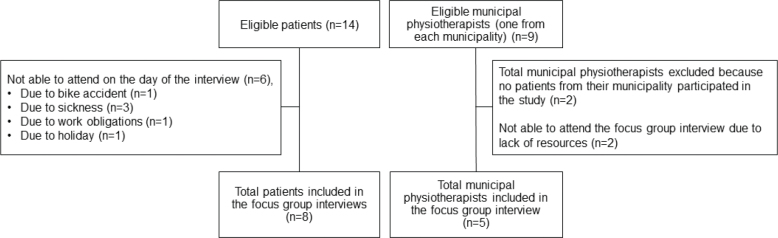
Study enrolment flowchart.

**Fig. 2:**
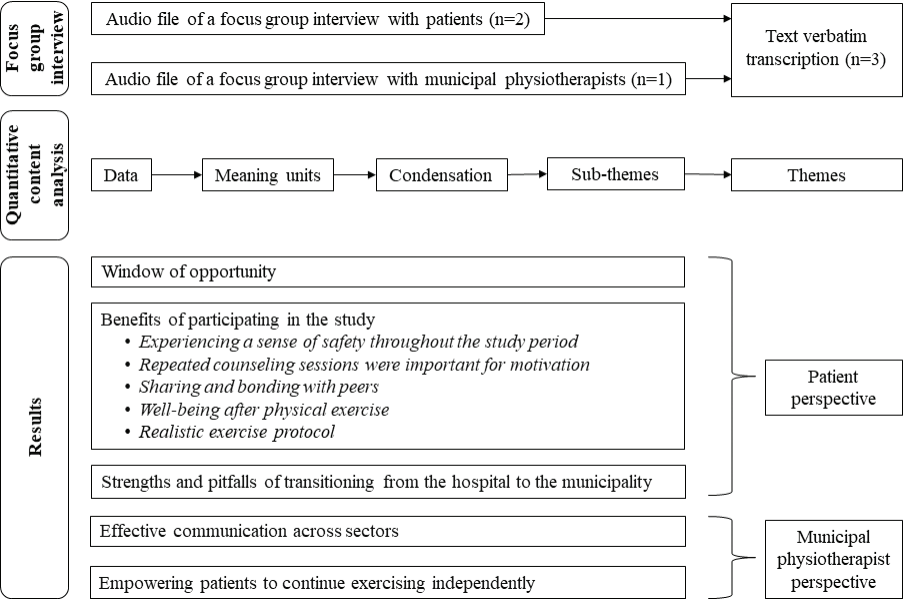
Flowchart of methodology and results.

The focus-group interviews took place from December 2022 to January 2024 and included 13 informants (Fig. 1). Six patients were unable to attend on the day of the interview for various reasons, and 2 municipalities lacked the resources to send a physiotherapist on the day of the interview (Fig. 1). The focus-group interviews were subsequently conducted with 3–5 informants in each interview. The baseline characteristics of all informants are described in Table III. The data analysis identified 5 categories: the first 3 categories represent the patients’ perspectives, and the remaining 2 the perspectives of the municipal physiotherapists: (*i*) window of opportunity, (*ii*) benefits of participating in the study, (*iii*) strengths and pitfalls of transitioning from the hospital to the municipalities, (*iv*) effective communication across sectors, and (*v*) empowering patients to continue exercising independently (Fig. 2).

**Table III:** Baseline characteristics of patients and municipal physiotherapists

Variables	
Patients with stroke	(*n* = 8)
Male sex, *n* (%)	7 (88)
Ethnicity, Caucasian, *n* (%)	8 (100)
Age, mean years (SD) [range]	64 (6) [58–72]
Time from stroke onset to exercise initiation, days, mean [range]	23 [12–76]
Mobility	
Outside ambulation without aids on discharge, *n* (%)	8 (100)
NIHSS (National Institutes of Health Stroke Scale), mean points (SD)	0.9 (1.1)
Education	
Apprenticeship/high school or short-cycle tertiary education (1–2 years), *n* (%)	3 (37.5)
Bachelor, Master’s or higher education, *n* (%)	5 (62.5)
Marital status	
Cohabitants, *n* (%)	7 (87.5)
Working status	
Working, *n* (%)	5 (62.5)
Retired, *n* (%)	3 (37.5)
Physiotherapists working at community rehabilitation centres	(*n* = 5)
Working experience, mean years (SD) [range]	14 (9) [6–30]
Male sex, *n* (%)	2 (40)

### Window of opportunity

Being invited into the study in the immediate aftermath of the stroke and during hospitalization was important for the patients’ motivation to participate in The Stroke School, even though the study information was overwhelming at the early inclusion. The vulnerability brought on by having a stroke motivated patients to engage in the study to prevent recurrence. Had they been asked after hospital discharge, the patients hypothesized that there would probably have been many competing issues to handle regarding reorganizing their daily lives. The patients appreciated that The Stroke School was initiated closely after their hospital discharge rather than waiting for weeks before a sufficient number of patients were included to establish an exercise group.

*I think the motivation [for participation] is highest [early after stroke onset] when they [the health professionals] tell you how bad it is … [the stroke] … and say if you do this [participate in the study] you may eliminate the chance of recurrence. (Male patient, 69 years old, retired)*

### Benefits of participating in the study

Patients described several benefits from participating in the study.

*(a) Experiencing a sense of safety throughout the study period*. Participating in The Stroke School instilled a sense of safety in patients and their families because they were followed closely for a year with physical examinations, questionnaires on stroke sequelae, routine medical examinations, individual stroke prevention sessions, and individual follow-up sessions. Being closely monitored throughout the study reassured patients and their families that it was safe to be physically active. At the same time, they felt acknowledged and understood, which further encouraged them to continue being physically active. Initially, they were apprehensive about the cardiopulmonary exercises where they could hear their heartbeat and became fearful that exercising would result in a recurrent stroke. Once they started exercising under supervision this fear subsided.

*Yes in the beginning I was worried about increasing my heart rate … I thought it was dangerous for me…. I was afraid that being physically active would result in a recurrent stroke…. That is all history now. (Male patient, 60 years old, on sick leave)*

*(b) Repeated counselling sessions were important for motivation.* During the study, the patients appreciated having multiple opportunities to talk with health professionals about stroke prevention and healthy lifestyle changes: first, at the hospital with the primary researcher, and, second, with the municipal physiotherapists. One advantage was the repetition of information that might otherwise have been forgotten. Another advantage was that some patients became more motivated for lifestyle changes later in the rehabilitation phase as they regained mental surplus.

I think it is great that there is a prolonged study period [several occasions] … where you can ask your questions to different health professionals during the study. (Male patient, 60 years old, on sick leave)

*(c) Sharing and bonding with peers* The patients highlighted the social benefits of exercising with peers. Having a stroke was a shared experience, and they appreciated the opportunity to confide with like-minded individuals before and after the exercise sessions. The themes they discussed were how to deal with specific stroke symptoms, their experience with preventive medication, how to talk with their spouse or relatives about having a stroke, or how to return to work.

*We all have the experience of having a stroke…. [I liked] having the opportunity to talk [with peers] for 5–10 minutes before or/and after the exercise sessions. To talk about our experiences with everyday life with stroke. (Male patient (A), 58 years old, working)*

They also felt more obligated by being part of a group, making it more difficult to miss out on an exercise session when other peers were expecting you. They also pushed themselves harder when exercising in groups, compared with exercising individually. They described the atmosphere during the hospital exercise sessions as light and humorous with room for competition.

*(d) Well-being after physical exercise.* The patients described the physical well-being they experienced during and after exercising. Although they felt tired immediately after exercising, they felt re-energized in the following days and committed to continue exercising.

*… you are exhausted right after the exercise session … but already the next day and the following days you feel re-energized. (Male patient (B), 58 years old, working)*

*(e) Realistic exercise protocol.* The minimal stroke symptoms experienced by the patients allowed them to engage in biking exercises, which promoted self-reliance and facilitated the initiation of healthy habits. The Stroke School’s exercise routine was praised for its simplicity, sparing patients from spending mental energy on thinking up exercises. Also, the municipal physiotherapists experienced that the patients completed the exercise protocol.

*… yes, I think it [the exercise protocol] seemed realistic … at least for the patients we have seen. Of course, it is exhausting … but it has to be [with this level of intensity]…. I think they seemed comfortable going through with it. (Female physiotherapist, 10 years’ work experience)*

### Strengths and pitfalls of transitioning from the hospital to the municipalities

The patients acknowledged the logic behind switching from the hospital to the municipality. However, they would prefer all exercise sessions to be at the hospital, if given the choice. They found that the municipalities could not schedule the exercise sessions out-of-hours to accommodate work obligations as was done at the hospital. Furthermore, they felt it was motivating to exercise with health professionals who biked along with and encouraged them. They also experienced they were given less attention in the municipality sessions, as they exercised in sessions dedicated to patients with cardiac conditions. Switching to the municipality gave them a feeling of starting from scratch with new peers and health professionals, and a new exercise schedule requiring them to reorganize their week. On the positive side, it was convenient that they could exercise close to home with minimal transport. All patients felt welcome and were greeted warmly by the municipal physiotherapists. The municipalities invited patients to exercise with the cardiac rehabilitation group in the last half of each session where they enjoyed engaging in new modalities of exercise. This prevented boredom and eased their integration into the cardiac group.

*For me who never participated in leisure sport … to try out different exercise modalities and thereby be inspired to continue exercising [after study termination] was good…. I fear that when this study terminates, the couch will be too appealing…. I would prefer to continue exercising, as it makes me feel better. (Male patient, 60 years old, on sick leave)*

In the municipality, the patients completed their pre-defined cardiovascular exercise programme individually on stationary bicycles during the first half of their exercise session (this part of their programme differed from that of the cardiac group). During this time, they felt left to themselves and had to push themselves to reach the required intensity zone.

*… no, we have not been integrated [with the cardiac rehabilitation group in the municipality] … because our life situation is different from the patients with cardiac problems…. But they [the physiotherapists] have kept an eye on me. (Male patient (A), 58 years old, working)*

### Effective communication across sectors

The municipal physiotherapists felt well informed and prepared for their role, and they enjoyed being part of a research study. Easy access to the lead researchers strengthened collaboration across sectors. A general standard electronic reference (REF15) was implemented to notify the municipality about new patients along with an e-mail (including patient information) sent directly to the physiotherapist involved.

*It was exciting to be part of a research study and to follow a strict exercise protocol … as we are not used to this with our other patient groups in the municipality. (Male physiotherapist, 6 years’ work experience).*

*I think we were well informed about who was coming and when they were coming. That made it [our job] easy. (Male physiotherapist, 10 years’ work experience)*

The municipal physiotherapists found it easy to document the patients’ progress in REDcap (Research Electronic Data Capture – a secure web application for managing online databases; https://project-redcap.org/).

*We collected and entered data [in REDcap] for one week at a time…. It worked well with the [written REDcap] manual we have received…. I think it was fine, once you were logged in. (Male physiotherapist, 10 years’ work experience)*

### Empowering patients to continue exercising independently

Encouraging the patients to exercise after study termination was a challenging task for the physiotherapists. This was especially the case with patients who had not previously engaged regularly in physical exercise before study participation and those who suffered from post-stroke fatigue.

*What is challenging, is also a challenge with other patients … we often discuss [in general between colleagues], whether it makes a difference to increase the functional capacity of our patients for three months if they stop exercising right after. (Female physiotherapist, 16 years’ work experience)*

The physiotherapists tried to motivate patients individually when they started exercising in the municipality. After each exercise session, they emphasized the importance of exercising regularly and used different approaches to motivate the patients. This included varying exercise modalities, differentiating between individual/group exercise, considering whether to exercise alone or with family/friends, and reflecting on the time of the day to exercise. Approaches could also include verbalizing that physical exercise can be an everyday activity, easily integrated into their daily living. Having several individual follow-up sessions to motivate and show that they cared for the patients was important. Their main objective was to encourage patients to be accountable to themselves rather than to the physiotherapist.

*… the most important thing is that they are accountable to themselves [and that they take charge]. (Female physiotherapist, 30 years’ work experience)*

## DISCUSSION

This qualitative study identified that both patients and municipal physiotherapists took great value in participating in The Stroke School. The patients reported a peak in motivation to engage in exercise when included closely following their stroke diagnosis, as they wished to take quick action on stroke prevention. A sense of safety during the study was reported by the patients as they had regular follow-ups on physical examinations and repeated counselling sessions. Participating in an achievable intervention for the patients resulted in a subsequent state of natural well-being, and the patients profited socially from exercising with peers. Benefiting from peer support was more challenging when exercising within the municipality, where patients felt somewhat more on their own. The physiotherapists experienced effective communication and collaboration across sectors but encountered difficulty in empowering the patients to exercise independently after termination of the study intervention. The findings highlight key elements for amending minor administrative study procedures when paving the way for the subsequent randomized controlled trial.

We found that early-initiated exercise was important for the patients as the stroke diagnosis caused a state of vulnerability with a concomitant high motivation to prevent recurrence. At present, patients with minor strokes and TIA are offered only preventive medication but no systematic rehabilitation. In contrast, patients with myocardial infarctions have systematically been offered cardiac rehabilitation (in the local municipality) for many years, including supervised exercise twice a week and counselling sessions on secondary prevention (15). This discrepancy between patient groups who both have an increased risk of recurrent events, if not supported in reducing well-known modifiable risk factors, is untenable. Patients with myocardial infarction completing cardiac rehabilitation achieved a risk reduction in acute hospital admissions from 31% to 26% compared with those who did not attend cardiac rehabilitation (16). By inviting patients with minor stroke or TIA to attend The Stroke School with cardiovascular exercise, we hope to sustain the initiated physical activity beyond the 6 months previously seen (17), and thereby achieve a reduction in recurrence and hospitalization along with improvements in exercise capacity, quality of life, and psychological well-being (16).

Our findings on barriers and motivators for physical activity after stroke are consistent with previous research (7). In both studies, patients appreciated local exercise to reduce transport, group-based exercise with peers, and supervision from health professionals, all conditions that promoted patient safety. Both studies found that, if exercise could be a part of patients’ daily routines, it would become natural for them, and they would have fewer second thoughts about exercising. Despite being aware of the motivational factors associated with physical activity following a stroke, patients ceased to engage in physical activity 1 year after their stroke (17). This highlights the challenges associated with maintaining long-term behavioural changes. The participating municipal physiotherapists found it difficult to motivate the patients to keep exercising after the termination of the study. Life-long maintenance of exercise post-stroke is crucial to upholding physical function, preventing cardiovascular risk factors, and reducing hospitalization (1). The patients were encouraged to commit to a healthy lifestyle and to follow the World Health Organization’s (WHO) recommended level of physical activity for adults ≥ 65 years of age. The WHO recommendations include a weekly minimum of 150 min of moderate-intensity activity, 75 min of vigorous-intensity activity, or an equivalent combination of the two (18). In studies on patients with cardiovascular disease, it is also challenging to maintain long-term physical activity without continued supervision (19, 20). In cardiac rehabilitation, patients were taught various exercises to adopt a long-term physical routine, but maintaining an exercise routine was difficult, resulting in the loss of the acquired health benefits (19). Only 25–40% of cardiac patients remained physically active 1 year after cardiac rehabilitation (21, 22). Likewise, patients with lacunar stroke included in a randomized controlled study on high-intensity training with 1-year follow-up failed to continue the initial level of exercise, as the significant increase in vigorous-intensity activity observed at 6-month follow-ups was not maintained 1 year post-stroke (17). In cardiovascular diseases, using individual text message-based mobile health interventions encouraged patients to increase physical activity after cardiac rehabilitation (23). According to a systematic review, individual text messages may be interpreted with caution due to the low quality of evidence, the small sample size, and the need to measure the effect across various diagnoses of sedentary behaviour (24). Studies identifying effective nudging techniques to improve long-term physical activity are highly warranted, and also in patients with minor strokes.

The patients included in this study expressed the significance of establishing bonds with peers as a motivating element in effecting lifestyle modifications post-stroke. This interpersonal connection fostered an exchange of ideas and cultivated shared comprehension among peers. In a 10-week self-management programme tailored for patients with stroke and their partners, peer support emerged as the most valued aspect among patients, emphasizing peer support as a central factor in improving rehabilitation adherence and experience (25). Moreover, interventions incorporating peer support for patients with stroke have demonstrated potential benefits in terms of both physical and psychological outcomes by heightening the sense of accountability and motivation (25). Another method, peer-led peer support, improves social communication better than staff-led initiatives by fostering empathetic connections among those with similar life experiences (26). A qualitative study on stroke patients found that peer-led peer support enhanced self-reliance and autonomy by allowing patients to explore their capabilities (26). Nonetheless, further investigation is warranted to elucidate the impact of peer support interventions on social participation and their subsequent influence on quality of life (27).

### Strengths and limitations

A strength of the study was the transparent and detailed description of the study design and its context throughout the study (13), including an audit trail made by NVivo (https://lumivero.com/), and reporting according to the COREQ checklist (10). The trustworthiness of the findings was increased by investigator triangulation in the analysis of data (13). Another strength of the study was the variation in patients included regarding age and employment status. Also, the physiotherapists represented both males and females with various lengths of work experience, which provided insight into The Stroke School procedure from different perspectives. The results were supported by citations from patients and physiotherapists, though this selection may be biased. We provided a relaxed atmosphere during the focus-group interviews by introducing one another, and by expressing a genuine interest in their opinion through our tone of voice and body language. This resulted in all patients and physiotherapists having their say in a reflective and eager dialogue with each other. The sample included only patients who had completed the supervised Stroke School intervention and were willing to participate in the interviews. The pre-established relationships between the patients and the researchers and the physiotherapists and researchers may have affected the answers due to a possible desire to please and reflect positively on The Stroke School design. We tried to avoid this by deciding *a priori* that the researcher who had examined and exercised with the respective patients would only act as an observer. Nevertheless, neither the patients nor the physiotherapist seemed conflicted about telling us if The Stroke School intervention lacked structure or was exhausting to complete. The results of a qualitative study cannot be statistically generalized, but they may be transferable to a similar population, in a cultural, organizational, and economic context (28).

### Conclusion

This cross-sectoral exercise intervention helped to ensure patients’ sense of safety through frequent exercise sessions, follow-up examinations, and repeated counselling sessions. Patients were highly motivated to exercise immediately after their stroke diagnosis. The municipal physiotherapists appreciated effective communication across sectors, leading to fruitful collaboration. The study highlights a continued need for research on how to facilitate life-long exercise behaviour changes.

## References

[1] Saunders DH, Sanderson M, Hayes S, Johnson L, Kramer S, Carter DD, et al. Physical fitness training for stroke patients. Cochrane Database Syst Rev 2020; 3: Cd003316. 10.1002/14651858.CD003316.pub7 32196635 PMC7083515

[2] Saunders DH, Sanderson M, Hayes S, Kilrane M, Greig CA, Brazzelli M, et al. Physical fitness training for stroke patients. Cochrane Database Syst Rev 2016; 3: CD003316. 10.1002/14651858.CD003316.pub6 27010219 PMC6464717

[3] Newitt R, Barnett F, Crowe M. Understanding factors that influence participation in physical activity among people with a neuromusculoskeletal condition: a review of qualitative studies. Disabil Rehabil 2016; 38: 1–10. 10.3109/09638288.2014.996676 25586798

[4] Nicholson S, Sniehotta FF, van Wijck F, Greig CA, Johnston M, McMurdo ME, et al. A systematic review of perceived barriers and motivators to physical activity after stroke. Int J Stroke 2013; 8: 357–364. 10.1111/j.1747-4949.2012.00880.x 22974010

[5] Faulkner J, McGonigal G, Woolley B, Stoner L, Wong L, Lambrick D. A randomized controlled trial to assess the psychosocial effects of early exercise engagement in patients diagnosed with transient ischaemic attack and mild, non-disabling stroke. Clin Rehabil 2015; 29: 783–794. 10.1177/0269215514555729 25352617

[6] Faulkner J, Lambrick D, Woolley B, Stoner L, Wong LK, McGonigal G. Effects of early exercise engagement on vascular risk in patients with transient ischemic attack and nondisabling stroke. J Stroke Cerebrovasc Dis 2013; 22: e388–96. 10.1016/j.jstrokecerebrovasdis.2013.04.014 23680679

[7] Krawcyk RS, Christoffersen LC, Danielsen AK, Kruuse C. Motivators for physical activity in patients with minor stroke: a qualitative study. Disabil Rehabil 2023; 45: 277–285. 10.1080/09638288.2022.2032409 35119324

[8] Avery KN, Williamson PR, Gamble C, O’Connell Francischetto E, Metcalfe C, Davidson P, et al. Informing efficient randomised controlled trials: exploration of challenges in developing progression criteria for internal pilot studies. BMJ Open 2017; 7: e013537. 10.1136/bmjopen-2016-013537 PMC5318608 28213598

[9] Kjærgaard Danielsen A, Spanager L. The use of focus group interviewing within health sciences. Dan Med J 2012; 174: 1298–1302. 22564686

[10] Tong A, Sainsbury P, Craig J. Consolidated criteria for reporting qualitative research (COREQ): a 32-item checklist for interviews and focus groups. Int J Qual Health Care 2007; 19: 349–357. 10.1093/intqhc/mzm042 17872937

[11] Carlsen B, Glenton C. What about N? A methodological study of sample-size reporting in focus group studies. BMC Med Res Methodol 2011; 11: 26. 10.1186/1471-2288-11-26 21396104 PMC3061958

[12] Elo S, Kyngäs H. The qualitative content analysis process. J Adv Nurs 2008; 62: 107–115. 10.1111/j.1365-2648.2007.04569.x 18352969

[13] Korstjens I, Moser A. Series: Practical guidance to qualitative research. Part 4: Trustworthiness and publishing. Eur J Gen Pract 2018; 24: 120–124. 10.1080/13814788.2017.1375092 29202616 PMC8816392

[14] World Medical Association Declaration of Helsinki: ethical principles for medical research involving human subjects. JAMA 2013; 310: 2191–2194. 10.1001/jama.2013.281053 24141714

[15] BACPR. Cardiovascular disease prevention and rehabilitation 2023. 4th ed. London: British Association for Cardiovascular Prevention and Rehabilitation; 2023.

[16] Dalal HM, Doherty P, Taylor RS. Cardiac rehabilitation. BMJ 2015; 351: h5000. 10.1136/bmj.h5000 26419744 PMC4586722

[17] Krawcyk RS, Vinther A, Petersen NC, Faber J, Iversen HK, Christensen T, et al. High-intensity training in patients with lacunar stroke: a one-year follow-up. J Stroke Cerebrovasc Dis 2023; 32: 106973. 10.1016/j.jstrokecerebrovasdis.2022.106973 36623990

[18] Bull FC, Al-Ansari SS, Biddle S, Borodulin K, Buman MP, Cardon G, et al. World Health Organization 2020 guidelines on physical activity and sedentary behaviour. Br J Sports Med 2020; 54: 1451–1462. 10.1136/bjsports-2020-102955 33239350 PMC7719906

[19] Graham H, Prue-Owens K, Kirby J, Ramesh M. Systematic review of interventions designed to maintain or increase physical activity post-cardiac rehabilitation phase II. Rehabil Process Outcome 2020; 9: 1179572720941833. 10.1177/1179572720941833 34497468 PMC8282140

[20] Dolansky MA, Stepanczuk B, Charvat JM, Moore SM. Women’s and men’s exercise adherence after a cardiac event. Res Gerontol Nurs 2010; 3: 30–38. 10.3928/19404921-20090706-03 20128541 PMC2897096

[21] Guiraud T, Granger R, Gremeaux V, Bousquet M, Richard L, Soukarie L, et al. Accelerometer as a tool to assess sedentarity and adherence to physical activity recommendations after cardiac rehabilitation program. Ann Phys Rehabil Med 2012; 55: 312–321. 10.1016/j.rehab.2012.05.002 22742999

[22] Arrigo I, Brunner-LaRocca H, Lefkovits M, Pfisterer M, Hoffmann A. Comparative outcome one year after formal cardiac rehabilitation: the effects of a randomized intervention to improve exercise adherence. Eur J Cardiovasc Prev Rehabil 2008; 15: 306–311. 10.1097/HJR.0b013e3282f40e01 18525385

[23] Martinello N, Saunders S, Reid R. The effectiveness of interventions to maintain exercise and physical activity in post-cardiac rehabilitation populations: a systematic review and meta-analysis of randomized controlled trials. J Cardiopulm Rehabil Prev 2019; 39: 161–167. 10.1097/HCR.0000000000000404 31021997

[24] Patterson K, Davey R, Keegan R, Freene N. Smartphone applications for physical activity and sedentary behaviour change in people with cardiovascular disease: a systematic review and meta-analysis. PLoS One 2021; 16: e0258460. 10.1371/journal.pone.0258460 34634096 PMC8504773

[25] Tielemans NS, Schepers VP, Visser-Meily JM, van Haastregt JC, van Veen WJ, van Stralen HE, et al. Process evaluation of the Restore4stroke Self-Management intervention ‘Plan Ahead!’: a stroke-specific self-management intervention. Clin Rehabil 2016; 30: 1175–1185. 10.1177/0269215515620255 26658332 PMC5131629

[26] May C, Bieber K, Chow D, Mortenson WB, Schmidt J. Experiences of adults with stroke attending a peer-led peer-support group. Brain Impair 2023; 24: 443–455. 10.1071/IB23051 38167360

[27] Wan X, Chau JPC, Mou H, Liu X. Effects of peer support interventions on physical and psychosocial outcomes among stroke survivors: a systematic review and meta-analysis. Int J Nurs Stud 2021; 121: 104001. 10.1016/j.ijnurstu.2021.104001 34246069

[28] Malterud K. Qualitative research: standards, challenges, and guidelines. Lancet 2001; 358: 483–488. 10.1016/S0140-6736(01)05627-6 11513933

